# Association of Atopic Dermatitis with Depression and Suicide: A Two-Sample Mendelian Randomization Study

**DOI:** 10.1155/2022/4084121

**Published:** 2022-02-03

**Authors:** Hong-jiao Qi, Lin-Feng Li

**Affiliations:** Department of Dermatology, Beijing Friendship Hospital, Capital Medical University, Beijing, China

## Abstract

**Background:**

Atopic dermatitis (AD) has long been hypothesized to be associated with risk of depression and suicide, but the causal relationship between them is still unclear.

**Objective:**

To evaluate the causality between AD, depression, and suicide using a Mendelian randomization (MR) approach.

**Method:**

We extracted summary-level data for AD, major depression, and suicidal ideation or attempt from published, nonoverlapping genome-wide association studies (GWAS). Inverse variance-weighted (IVW) analysis was used as the primary analysis. Alternate methods, including weighted median, MR Egger, MR pleiotropy residual sum and outlier, weighted mode, and leave-out analysis, were performed to assess pleiotropy.

**Results:**

13 SNPs (13,287 cases and 41,345 controls) were selected as instrumental variables (IVs). The IVW analysis indicated a statistically significant but small causal effect of AD on major depression (OR = 1.027, 95% CI 1.004-1.050; *p* = 0.020). No significant evidence was observed for a causal effect of AD on suicide. No significant effect of pleiotropy was found.

**Conclusion:**

AD has a significant but small effect on major depression, but not on suicide.

## 1. Introduction

Atopic dermatitis (AD, eczema, and atopic eczema) is one of the most common chronic, inflammatory, relapsing, skin diseases [1]. Up to 17.1% of adults and 22.6% of children are diagnosed with AD each year [2]. It is characterized by eczematous lesions, intense pruritus, and a chronic or relapsing disease course [3]. AD largely decreases health-related quality of life (HRQoL) and mental health level [4, 5]. In addition, work productivity and activities of adult patients are impaired by the presence of AD [6, 7]. Furthermore, considerable economic costs also represent a burden to the affected individual and society [8, 9].

Over the last few years, an increasing number of studies aimed at the associations between AD and mental disorders, especially depression and suicide, have been widely researched. However, the results of previous studies have been mixed. Some studies have shown a positive correlation between AD and depression [5, 6, 10–19], while other studies did not find a significant association between depression and AD [20–23]. Similarly, with regard to the link between AD and suicide, the previous results are conflicting [15, 24–26]. Differences in population, sample size, and measurements of AD, depression, and suicide may have contributed to these conflicting findings. Besides, current evidence on the associations between AD, depression, and suicide is mainly based on population-based observational studies, which are susceptible to potential confounders and inverse causation. The causality between AD, depression, and suicide is still unclear.

In order to evaluate causality between AD, depression, and suicide, additional methods are needed. Mendelian randomization (MR) analysis is a novel epidemiological method to assess the causation between an exposure and an outcome [27], with less susceptibility to potential confounders and reverse causation by using genetic variants as instrumental variables (IVs) [28]. Taking advantage of the random allocation of genes which occurs at meiosis, MR is analogous to randomized clinical trials [29]. MR analysis uses genetic data (i.e., single-nucleotide polymorphisms (SNPs)) as genetic instruments to estimate the effect of concerned exposure on an outcome [30]. Results from an MR analysis may provide strong evidence for causality, if the genetic instruments used are associated with the exposure, only affect the outcome via the exposure, and are not associated with any of the potential confounders of the exposure [31, 32]. Specifically, because the genotype is not modifiable by disease, this method avoids the bias of reverse causality [28].

Thus, we conducted a two-sample MR study to explore the causal associations between AD, depression, and suicide.

## 2. Methods

Our study was a secondary analysis of the publicly available data. Informed patients' consent and ethical approvals were obtained by original GWAS studies; no additional ethics approval or consent to participate was required.

### 2.1. Study Design

We conducted a 2-sample MR study to investigate causal relationship between AD, depression, and suicide. Single-nucleotide polymorphisms (SNPs) selected as instruments for risk factors are randomly allocated and are therefore less likely to be affected by confounding or reverse causation [33]. Regarding natural randomization, the MR approach imitates a randomized controlled trial based on summary-level data from observational studies [31].

### 2.2. Data Sources and Selection of SNPs

Genetic associations with AD were obtained from the publicly available GWAS among individuals of mostly European ancestry contributed from the EArly Genetics and Lifecourse Epidemiology (EAGLE) eczema consortium [34]. Summary-level data from a meta-analysis using 26 genome-wide association studies (GWASs) include 13,287 cases and 41.345 controls [34]. AD was diagnosed by self-report questionnaire or interview with a doctor. Genetic association data on major depression was obtained from three largest GWAS of major depression, including 246,363 cases and 561,190 controls of mostly European ancestry. Major depression was confirmed by self-reported measurements or medical records [35]. GWAS summary statistics for suicidal ideation or attempt derived from UK biobank which comprise 1986 cases and 369930 controls of European ancestry [36]. Thirteen SNPs were identified as associated with atopic dermatitis with genome-wide significance (*p* < 5 × 10^−8^), with independent inheritance (*r*^2^ was set as <0.01 to include more SNPs as the IV), and without linkage disequilibrium (LD) in summary statistics. All of these 13 SNPs were available in the summary data for major depression and suicidal ideation or attempt. Details of the included SNPs are shown in Tables S1 and Tables S2, respectively. We also applied *r*^2^ < 0.001 as LD threshold to evaluate possible bias caused by selection of SNP.

### 2.3. Statistical Analysis

Inverse variance-weighted (IVW) linear regression was used as the primary analysis for the associations between AD and depression. IVW assumes that all instruments are valid and that there is no horizontal pleiotropy (i.e., SNPs are associated with the outcome exclusively via the exposure) [37, 38]. In addition, weighted median approach [38], MR Egger regression [39], weighted mode [40], and MR pleiotropy residual sum and outlier (MR PRESSO) [41] were also performed to complement IVW estimates as these approaches could provide more robust estimates in a broader set of scenarios but are less efficient (wider confidence interval (CI)).

Several sensitivity analyses were used to detect underlying pleiotropy, and the heterogeneity for MR estimates can be severely violated. We used Cochran *Q* test from the IVW approach to assess potential horizontal pleiotropy, *p* < 0.05 was considered the presence of horizontal pleiotropy. The intercept obtained from the MR Egger regression was an indicator for directional pleiotropy. Leave-one-out analysis was also performed to evaluate whether the MR estimate was driven or biased by a single SNP. The statistical power was estimated using mRnd [42], and other statistical analyses were performed using R software 4.0.3. Statistical significance was established with 2-sided tests with *α* = 0.05.

## 3. Results

### 3.1. The Effect of AD on Major Depression

**Figure 1:**
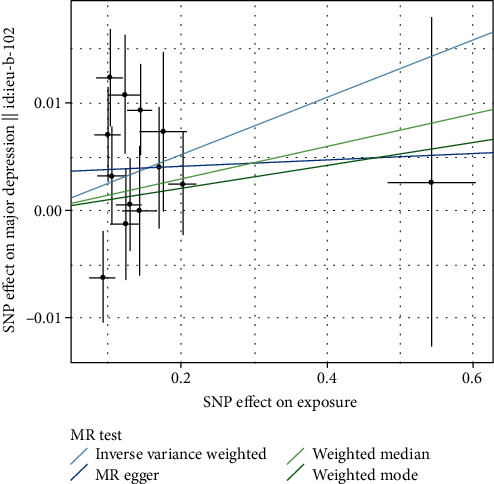
Mendelian randomization analysis for AD on risk of major depression.

We observed a significant causal effect of AD on major depression using IVW analysis (odds ratio (OR) = 1.027, 95% CI 1.004-1.050; *p* = 0.020). MR PRESSO also showed significant evidence (OR = 1.027, 95% CI 1.004-1.050; *p* = 0.038). The association was consistent across weighted median method, MR Egger, and weighted mode analyses, although non-significant (Table 1 and Figure 1).

**Figure 2:**
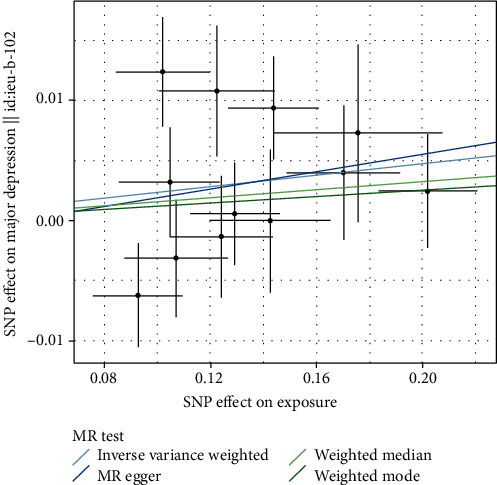
Mendelian randomization analysis for AD on risk of major depression.

When applied 0.001 as the threshold of LD, 12 SNPs were selected as IV. No significant evidence was found for a causal effect of AD on major depression, using the IVW analysis (OR = 1.024, 95% CI 0.999-1.050; *p* = 0.058) and other methods (Table 2 and Figure 2).

Leave-one-out analysis was performed to identify potential influencing SNPs that could bias the causal association. Pooled results were consistent after omitting single SNPs, indicating no influence of SNP on the causal association. *p* values for Cochrane *Q* test and MR Egger intercept were 0.181 and 0.466, respectively, suggesting no evidence of potential horizontal pleiotropy and heterogeneity.

### 3.2. The Effect of AD on Suicidal Ideation or Attempt

**Figure 3:**
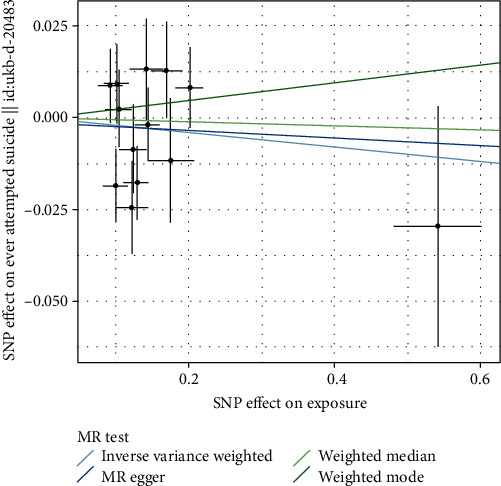
Mendelian randomization analysis for AD on risk of suicidal ideation or attempt.

No significant evidence was found for a causal effect of AD on suicidal ideation or attempt, using the IVW analysis (OR = 0.887, 95% CI 0.751-1.048; *p* = 0.158). The results neither weighted median, MR Egger, weighted mode, nor MR PRESSO analyses were significant (Table 3 and Figure 3).

Leave-one-out analysis showed no influence of SNP on the risk estimation of AD on suicidal ideation or attempt. *p* values for Cochrane *Q* test and MR Egger intercept were 0.148 and 0.666, respectively, suggesting no evidence of potential horizontal pleiotropy and heterogeneity.

## 4. Discussion

This study supported a small causal effect of AD on major depression, but not on suicide. However, the magnitude of the effect of AD on major depression was very small with an OR of 1.03, and the significance disappeared when stricter threshold for selection of SNPs was applied, which indicated that the effect of AD on major depression is limited, and caution should be taken when interpreted this result. In addition, this small effect might also limit the clinical implication of this finding.

Previous observational studies on the link between AD and depression have been controversial. The latest retrospective case-control study, which included 7,061 cases with depression and 7,061 matched controls without depression, found AD cases were significantly associated with depression in children and adolescents in Germany [10]. Treudler et al. got the results from a cross-sectional population-based study; when compared with controls, it showed that subjects with AD had higher scores for depressive symptoms (9.3% vs. 6.3%; *p* < 0.001) [11]. In a cross-sectional study of US adults, Chiesa Fuxench et al. reported that depression was 13.98% in the AD group versus 5.99% in the control group (*p* < 0.003) [5]. Silverberg et al. reported adults with AD vs. those without AD had higher mean Hospital Anxiety and Depression Scale depression (HADS-D) (6.0 vs. 4.3) scores and higher prevalences of abnormal HADS-D (13.5% vs. 9.0%) scores from a cross-sectional, population-based study in the US [12]. In a cross-sectional study, Eckert et al. found that depression was more prevalent in patients with AD, affecting 25.8% and 36.2% of those with AD and inadequately controlled AD, respectively, compared with 12.9% of controls without AD in European adults (*p* < 0.001 for both comparisons) [6]. However, other researchers reported depression is not significantly related to AD. Vittrup et al. observed no association with a hospital diagnosis of depression and AD in Danish children (aHR = 0.58, 95% CI: 0.21–1.56) [20]. Schut et al. reported depression was not significantly related to itch induced by the experimental video from their study (*r* = 0.069, *p* = 0.756) [23]. A Korean cross-sectional study showed the incidence of depression was not significantly different between AD and non-AD patients. Only severe AD showed a high OR of depression (moderate AD OR = 1.75; severe AD OR = 3.15; *p* < 0.0001) [20]. A case-control study which was conducted in a city in western Turkey revealed there is not a significant relation between AD and depression [22]. In a Mendelian randomization study conducted by Baurecht et al., no significant association between AD and depression was found [43]. The differences between their results and ours might result from the threshold of LD we used. Using a relatively wider threshold could filter more SNPs to strengthen the IVs. The differences also indicated the association between AD and depression still needed further studies to validate.

Results from the preceding studies on the association between AD and suicide have been conflicting as well. Thyssen et al. reported AD patients had an increased prevalence of suicidal ideation than non-AD subjects in Danish population [15]. In a Korean cross-sectional study, Huh et al. found after adjusting for confounding variables that atopic dermatitis was associated with higher risks of suicidal thoughts (OR = 1.77, 95% CI: 1.15–2.70, *p* = 0.01) compared to no diagnosis [24]. According to another Korean cross-sectional study, there is a significantly increased risk of suicide ideation (OR = 1.26, 95% Cl: 1.16-1.36), suicide planning (OR = 1.28, 95% Cl: 1.14-1.44), and suicide attempt (OR = 1.29, 95% Cl: 1.13-1.49) [25]. A German cross-sectional study showed an increased risk of suicidal ideation; the prevalence of attempted suicide in patients with AD was 6.6% (control: 0%, *p* = 0.035) [26]. But Ahn et al. reported that there was no significant difference in suicidal ideation between AD patients and non-AD patients (OR = 0.90, 95% CI: 0.76-1.06 *p* = 0.2143) [20]. One large European multicenter study demonstrates that there was no significant difference in suicidal ideation between AD patients and the control (OR = 1.32, 95% CI: 0.75-2.33) [17].

The mechanism underlying the association between AD and depression is not fully elucidated. A possible mechanism is the effect of severe and constant pruritus [44]. The prevailing theory for chronic itch-induced depression is the neuroendocrine hypothesis, which indicates that the occurrence of depression is deeply relevant to the dysfunction of hypothalamus-pituitary-adrenal (HPA) axis [45, 46]. In addition, sleep disturbance caused by itches may further strengthen the impact of AD on mental diseases [47, 48]. Social stigmatization due to skin lesions also contribute to psychiatric disease [49, 50]. Furthermore, elevated proinflammatory cytokines may affect neurotransmitter synthesis and metabolism, potentially contributing to the pathogenesis of mental disorders in AD [51, 52]. Likewise, several possible biologic mechanisms have been proposed to explain the association between AD and suicide. A role of inflammation, pruritus, and sleep disturbance induced by itches and social stigmatization in suicide have also been proposed [53–55].

There are some limitations to the present study. First, the summary-level GWAS data we used were based mainly on people of European ancestry. Therefore, results in this study may not be applicable to other populations. Second, onset age and disease severity of AD might influence the association between AD, depression, and suicide, given that AD often arises in childhood [3]. However, limited by the data, we were not able to perform subgroup analyses by age and severity. Third, an important limitation for MR study is potential pleiotropy. In this study, we applied various MR approaches to test for potential pleiotropy, and no evidence of pleiotropy was observed. Fourth, the definitions of AD used in the data are a mixture of self-reported measurements and clinical diagnosis, which might cause biases. Furthermore, the GWAS data used for depression ranged from broad depression (self-reported help-seeking for problems with nerves, anxiety, tension, or depression), probable major depression (self-reported depressive symptoms with associated impairment), to major depression (identified from hospital admission records) [34], which might also have an influence on our results [57]. Fifth, given that depression and suicide may occur across the lifetime and most of the AD data cases were younger adults, more high-quality studies are needed, especially for other age groups.

## 5. Conclusion

In conclusion, AD might have a significant but small effect on major depression; cautions need to be taken when interpreting this result.

## Figures and Tables

**Table 1:** The causal effect of AD on major depression.

Method	OR (95% CI)	*p* value	*R* ^2^	Statistical power
IVW	1.027 (1.004-1.050)	0.020	0.628	0.56
Weighted median	1.015 (0.989-1.042)	0.267	0.319	0.13
MR egger	1.003 (0.940-1.070)	0.929	0.027	0.05
Weighted mode	1.011 (0.978-1.044)	0.539	0.173	0.07
MR PRESSO	1.027 (1.004-1.050)	0.038	0.574	0.53

CI: confidence interval; IVW: inverse variance-weighted; MR: Mendelian randomization; OR: odds ratio; SNP: single-nucleotide polymorphism.

**Table 2:** The causal effect of AD on major depression.

Method	OR (95% CI)	*p* value	*R* ^2^	Statistical power
IVW	1.024 (0.999-1.050)	0.058	0.556	0.44
Weighted median	1.016 (0.987-1.047)	0.282	0.337	0.16
MR egger	1.037 (0.929-1.157)	0.533	0.199	0.37
Weighted mode	1.013 (0.976-1.051)	0.512	0.210	0.09
MR PRESSO	1.024 (0.999-1.050)	0.085	0.514	0.41

**Table 3:** The causal effect of AD on suicidal ideation or attempt.

Method	OR (95% CI)	*p* value	No. of SNPs	*R* ^2^	Statistical power
IVW	0.887 (0.751-1.048)	0.158	13	0.413	1
Weighted median	0.869 (0.695-1.087)	0.218	13	0.380	1
MR egger	0.980 (0.611-1.571)	0.935	13	0.025	0.06
Weighted mode	0.860 (0.576-1.284)	0.476	13	0.215	1
MR PRESSO	0.887 (0.751-1.048)	0.184	13	0.391	1

CI: confidence interval; IVW: inverse variance-weighted; MR: Mendelian randomization; OR: odds ratio; SNP: single-nucleotide polymorphism.

## Data Availability

The data used to support the findings of this study are included within the article.

## Supplementary Materials

Supplementary Materials Table S1 Leave out analysis for the association between AD and major depression. Table S2 Leave out analysis for the association between AD and suicidal ideation or attempt.
