# Supplementary material for: Association of Atopic Dermatitis with Depression and Suicide: A Two-Sample Mendelian Randomization Study
Source: Biomed Res Int. 2022 Feb 3;2022:4084121. doi: 10.1155/2022/4084121 (PMC8831056; doi:10.1155/2022/4084121)
Supplement: Supplementary Materials — Table S1 Leave out analysis for the association between AD and major depression. Table S2 Leave out analysis for the association between AD and suicidal ideation or attempt. [file 4084121.f1.zip › 4084121.f1/Table S1 Leave out analysis 0719.docx]

Table S1 Leave out analysis for the association between AD and major depression.

| **Leave out analysis** |  |  |  |  |
| --- | --- | --- | --- | --- |
| SNP | OR | lci | uci | p |
| rs10790275 | 1.023583 | 1.000961 | 1.046715 | 0.040927 |
| rs12144049 | 1.029875 | 1.004147 | 1.056261 | 0.022569 |
| rs12188917 | 1.027129 | 1.002333 | 1.052538 | 0.031803 |
| rs12334935 | 1.031423 | 1.010585 | 1.052691 | 0.002968 |
| rs2212434 | 1.02901 | 1.004558 | 1.054058 | 0.019776 |
| rs2477121 | 1.024386 | 1.001007 | 1.04831 | 0.040808 |
| rs2918299 | 1.028426 | 1.004374 | 1.053053 | 0.020259 |
| rs4151657 | 1.021876 | 1.001586 | 1.042577 | 0.03444 |
| rs479844 | 1.021902 | 0.998494 | 1.045859 | 0.06688 |
| rs6062486 | 1.026599 | 1.002336 | 1.051449 | 0.031458 |
| rs61815704 | 1.029946 | 1.005148 | 1.055357 | 0.017647 |
| rs6419573 | 1.02917 | 1.00541 | 1.053492 | 0.015833 |
| rs8066625 | 1.025917 | 1.00171 | 1.05071 | 0.035712 |
| All | 1.026826 | 1.004165 | 1.049999 | 0.020071 |

OR: odds ratio; lcl: lower confidence intervals; ucl: upper confidence intervals
