# Supplementary material for: Association of Atopic Dermatitis with Depression and Suicide: A Two-Sample Mendelian Randomization Study
Source: Biomed Res Int. 2022 Feb 3;2022:4084121. doi: 10.1155/2022/4084121 (PMC8831056; doi:10.1155/2022/4084121)
Supplement: Supplementary Materials — Table S1 Leave out analysis for the association between AD and major depression. Table S2 Leave out analysis for the association between AD and suicidal ideation or attempt. [file 4084121.f1.zip › 4084121.f1/Table S2 Leave out analysis 0719.docx]

Table S2 Leave out analysis for the association between AD and suicidal ideation or attempt.

| **Leave out analysis** |  |  |  |  |
| --- | --- | --- | --- | --- |
| SNP | OR | lci | uci | p |
| rs10790275 | 0.879993 | 0.737478 | 1.050047 | 0.156122 |
| rs12144049 | 0.82238 | 0.702403 | 0.962851 | 0.015077 |
| rs12188917 | 0.927616 | 0.791291 | 1.087426 | 0.354179 |
| rs12334935 | 0.886916 | 0.742597 | 1.059283 | 0.185371 |
| rs2212434 | 0.927817 | 0.792507 | 1.08623 | 0.351566 |
| rs2477121 | 0.892429 | 0.747337 | 1.06569 | 0.208686 |
| rs2918299 | 0.886167 | 0.741195 | 1.059494 | 0.184863 |
| rs4151657 | 0.875947 | 0.735857 | 1.042706 | 0.136314 |
| rs479844 | 0.891982 | 0.742468 | 1.071605 | 0.222016 |
| rs6062486 | 0.900962 | 0.757381 | 1.071763 | 0.238991 |
| rs61815704 | 0.898513 | 0.745536 | 1.08288 | 0.261091 |
| rs6419573 | 0.882622 | 0.738726 | 1.054548 | 0.16911 |
| rs8066625 | 0.861053 | 0.732242 | 1.012524 | 0.070379 |
| All | 0.887201 | 0.751312 | 1.047668 | 0.158242 |

OR: odds ratio; lcl: lower confidence intervals; ucl: upper confidence intervals
